# A Galvanic Sensor for Monitoring the Corrosion Condition of the Concrete Reinforcing Steel: Relationship Between the Galvanic and the Corrosion Currents

**DOI:** 10.3390/s91108391

**Published:** 2009-10-26

**Authors:** Elsa Vaz Pereira, Rita Bacelar Figueira, Maria Manuela Lemos Salta, Inês Teodora Elias da Fonseca

**Affiliations:** 1 Laboratório Nacional de Engenharia Civil (LNEC), Av. do Brasil 101, 1700-066 Lisboa, Portugal; 2 Centro de Ciências Moleculares e Materiais (CCMM), DQB, Faculdade de Ciências, Universidade de Lisboa, Campo Grande Ed.C8, 1749-016 Lisboa, Portugal

**Keywords:** galvanic sensor, polarization resistance sensor, corrosion rate, reinforcing steel, carbonation, chloride ions

## Abstract

This work reports a study carried out on the design and performance of galvanic and polarization resistance sensors to be embedded in concrete systems for permanent monitoring of the corrosion condition of reinforcing steel, aiming to establish a correlation between the galvanic currents, *I*_gal_, and the corrosion currents, *I*_corr_, estimated from the polarization resistance, *R*_p_. Sensors have been tested in saturated Ca(OH)_2_ aqueous solutions, under a variety of conditions, simulating the most important parameters that can accelerate the corrosion of concrete reinforcing steel, such as carbonation, ingress of chloride ions, presence or absence of O_2_. For all the conditions, the influence of temperature (20 to 55 °C) has also been considered. From this study, it could be concluded that the galvanic currents are sensitive to the various parameters following a trend similar to that of the *R*_p_ values. A relationship between the galvanic and the corrosion current densities was obtained and the limiting values of the *I*_gal_, indicative of the state condition of the reinforcing steel for the designed sensor, were established.

## 1. Introduction

It is well known that steel passivation in concrete is due to the highly alkaline environment (pH: 12.5 to 13.6). However, steel passivity can be destroyed by local acidification, carbonation, ingress of chloride ions and/or depletion of O_2_, being the corrosion of reinforcements one of the major causes of the degradation of concrete structures in aggressive environments.

Structural deterioration of reinforced concrete structures affected by corrosion is a gradual process consisting of a few different phases during service life, including corrosion initiation, concrete cracking, excessive deflection and final collapse due to loss of structural strength.

In order to assist the development of reliable models that allow the design of new structures durable in aggressive environments and to establish rational maintenance and repair strategies of reinforced concrete structures affected by reinforcement corrosion, various systems for permanently monitoring the corrosion on site have been developed [1-12].

As well documented by Elsener [13] and others [14-20], electrochemical techniques (i.e. half-cell potential measurements, polarization resistance, potentiostatic and galvanostatic transients perturbations, electrochemical impedance spectroscopy, noise analysis, multielectrode systems, etc.) offer several advantages for reinforcement corrosion monitoring.

Song and Saraswathy [16] presented an exhaustive and well-documented review on the electrochemical techniques and sensors from the point of view of corrosion assessment and their application to civil engineering structures. McCarter and Vennesland [15] have also reviewed sensor systems for use in reinforced concrete systems. Zheng *et al.* [21] and Dickerson *et al.* [22] have published studies on the development of new permanent corrosion monitoring systems that provide relevant information on the rate of degradation of reinforced concrete in aggressive environments.

In the corrosion initiation period, when the aggressive agents penetrate the concrete cover until reaching the steel, the most relevant parameter is the chloride content, with the corrosion rate being identified as the most relevant parameter in the corrosion propagation period, during which the rebar corrodes until a maximum tolerable level of damage is reached [23].

Reinforcement corrosion rate has been evaluated continuously mainly by galvanic current and polarization resistance measurements. Galvanic macrocell sensors consisting of two dissimilar metals based on the well-known principles of galvanic corrosion were first proposed by Schiessl and Raupach [2]. The capability of these sensors to detect the initiation of corrosion is well-documented [2,3,7], however few studies have been performed on the ability of those sensors to estimate the instantaneous corrosion rate of the reinforcements [14,24].

A galvanic and a polarization resistance sensor to be embedded in concrete systems has been designed and built and its performance tested first in the laboratory, in solutions simulating concrete under aggressive conditions, and thereafter in new and repaired concrete for the evaluation of different surface treatments [5,25].

This paper reports a study on the developed sensors tested in saturated Ca(OH)_2_ aqueous solutions, under a variety of conditions simulating the most important parameters that can influence the corrosion of concrete reinforcing steel, such as carbonation leading to decreases of pH, ingress of chloride ions and the presence or absence of O_2_. For all the conditions, the influence of time and temperature (25 to 55 °C) has also been considered. A relationship between the galvanic currents and the corrosion rates of reinforcing steel, under a great variety of controlled laboratorial conditions, was established.

As emphasized by Martinez and Andrade in a recent paper [9], very few studies have been published on the *in-situ* monitoring of the corrosion rate, under the influence of natural climatic conditions. In fact, as it is well recognized by the authors, the environment at the surface of a rebar embedded in concrete can be significantly different from that seen under controlled laboratory conditions. Another study is now in progress, aiming to test and/or improve the established relationship between the galvanic currents and the corrosion rates of reinforcing steel, in concrete samples and in reinforced structures, using the developed sensors and external probes to measure the corrosion rate of the embedded rebar, under natural climatic conditions.

## 2. Experimental

**Figure 1.**
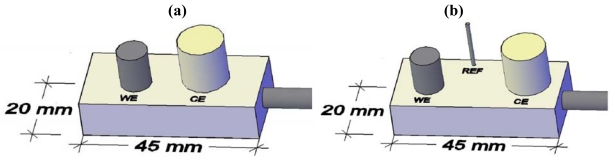
Schemes of the two sensors: (a) galvanic sensor: WE (steel); CE (stainless steel); (b) polarization resistance sensor; WE (steel); CE (stainless steel), RE (Ti/TiO_2_).

Two electrodes compose the galvanic sensor, *I*_gal_, the working electrode made of carbon steel and a stainless steel counter electrode [see Figure 1(a)]. The polarization resistance sensor, *R*_p_, presents a third electrode – an activated Ti wire acting as reference electrode [see scheme in Figure 1(b)]. These types of sensors can be used either in solution or in embedded concrete. The chemical composition of the reinforcing steel and of the stainless steel is given in Table 1.

**Figure 2.**
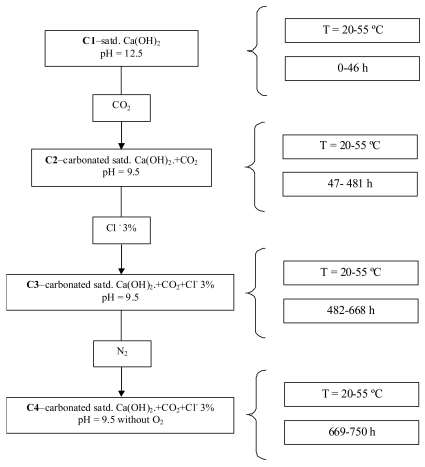
Scheme of the experimental conditions.

Saturated Ca(OH)_2_ aqueous solution, pH = 12.5, was used and then successively modified by bubbling CO_2_, followed by chloride ions addition (3%) and finally N_2_ bubbling for the removal of the dissolved O_2_. For each condition, different temperatures, ranging between 20 and 55 °C, have been considered. Figure 2 presents the scheme of the experimental conditions (C1 to C4).

**Figure 3.**
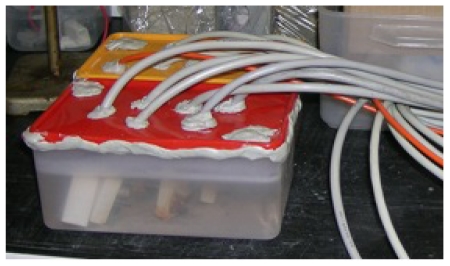
Photo of the PVC cell with the 12 sensors immersed in a thermostated solution.

For each condition (C1 to C4), six *R*_p_ sensors and six *I*_gal_ sensors were used. The polarization resistance, *R*_p_, was evaluated using the potentiostatic pulse method [5,20] with the polarization resistance, *R*_p_, calculated from the transients due to the application of a 10 mV anodic potential step for 100 s. The *R*_p_ measurements were performed periodically. A Voltalab PGZ 301 potentiostat was used, while the galvanic currents were acquired automatically every hour, using a data acquisition system, Datataker DT505. The temperature was controlled with a Hanna Instrument — HI 92840 C. All sensors were immersed in a closed PVC cell, under thermostatic conditions (see Figure 3).

## 3. Results and Discussion

**Figure 4.**
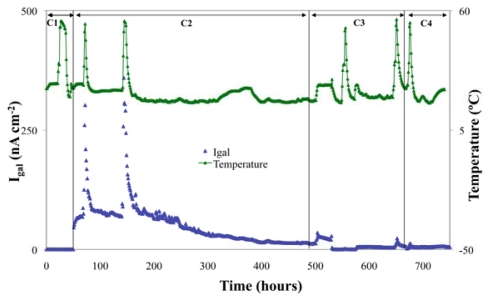
*I*_gal_ and temperature *vs.* immersion time for the conditions C1 to C4.

Figure 4 is a graphic representation of the average *I*_gal_ values of the steel working electrode, under the following experimental conditions: C1: satd. Ca(OH)_2_ solution, pH 12.5; C2: satd. Ca(OH)_2_ + CO_2_, pH 9.5; C3: satd. Ca(OH)_2_ + CO_2_ + 3% Cl; C4: satd. Ca(OH)_2_ + CO_2_ + 3% Cl^-^ + N_2_ (O_2_ depletion). For each condition, temperatures of 25, 35 and 55 °C have been set and the corresponding measurements have been performed.

For the steel in the passive state (condition C1), *I*_gal_ values < 0.1 nA cm^−2^ were obtained and no variation of *I*_gal_ with temperature was noticed. As the pH was lowered to 9.5 (condition C2), a passivity breakdown occurred and the process was sensitive to the temperature. A similar behavior was shown for the measurements corresponding to conditions C3 and C4 (*I*_gal_ rises with temperature). As expected, due to formation of the oxides in the working electrode surface, a decrease of *I*_gal_ with time was observed for conditions C2, C3 and C4.

**Figure 5.**
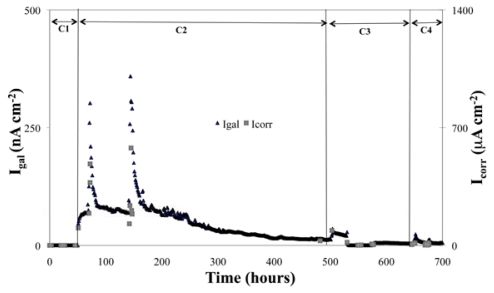
*I*_gal_ and *I*_corr_ as a function of immersion time, under the conditions C1 to C4.

Figure 5 gives the measured *I*_gal_ and the *I*_corr_ values estimated with the *R*_p_ and the Stern — Geary Equation: *I*_corr_ = B/*R*_p_ with B equal to 26 and 52 mV, used for the conditions of the passive and active state, respectively [20].

**Figure 6.**
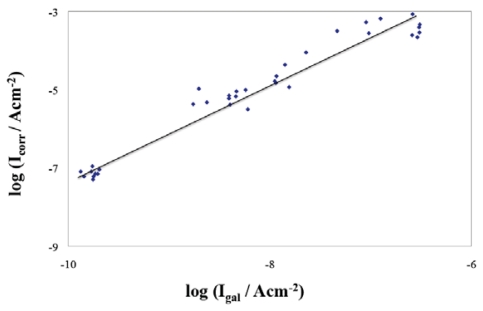
log *I*_gal_ *vs.* log *I*_corr_ plot.

Data show a similar trend of both values, in spite of its magnitude. It should be noted that the values of *I*_gal_ are currents related with the galvanic process, without further perturbation (free corrosion), while *I*_corr_ are currents resulting from small polarization. Figure 6 presents log *I*_gal_ *vs.* log *I*_corr_ plot, in order to obtain a relationship between both parameters. An almost linear relationship between log *I*_corr_ and log *I*_gal_, with a slope of *ca* 1.0, was obtained. The straight line in Figure 6 is described by the Equation: log *I*_corr_ (A cm^−2^) = 1.2 log *I*_gal_ (A cm^-2^) + 4.5, with r^2^ =0.957, which means: *I*_corr_ ≈ 10^9/2^ *I*_gal_^6/5^.

Corrosion current densities lower than 0.1 μA cm^−2^ have been reported as indicative of the reinforcing steel passive state, while currents higher than 1 μA cm^−2^ have been identified as corresponding to high corrosion rates [20,26]. If the relation *I*_corr_ ≈ 10^9/2^ *I*_gal_ ^6/5^ was applied to these values, equivalent limiting values using the galvanic currents measured with the proposed sensor could also be tentatively established. The corresponding values are given in Table 2.

## 4. Conclusions

In this work simple polarization resistance and galvanic sensors, suitable for embedding in concrete for the continuous monitoring of corrosion, were designed and tested in saturated Ca(OH)_2_ aqueous solutions, carbonated, with chloride addition and with O_2_ depletion, simulating the concrete pore solution.

All values corresponding to the conditions tested in this study have shown to obey the following relationship: *I*_corr_ ≈ 10^9/2^ *I*_gal_ ^6/5^. Taking it into account the limits of *I*_gal_ corresponding to the *I*_corr_ values defined in the literature, the values indicative of the condition of the reinforcing steel could be established as *I*_gal_ < 0.14 nA cm^−2^ corresponding to *I*_corr_ < 0.10 μA cm^−2^, indicative of the passive condition, and *I*_gal_ > 1 nA cm^−2^ corresponding to *I*_corr_ > 1 μA cm^−2^, indicative of the high corrosion rate.

The sensors have been tested in concrete pore solution under a great variety of controlled experimental conditions, and the limiting values of the *I*_gal_, indicative of the state condition of the corrosion state of the reinforcing steel, were established. In order to test the developed sensors and the correlation between *I*_gal_ and *I*_corr_ for the estimation of the corrosion rate in real systems, under the climatic conditions, studies in concrete samples and in reinforced structures are presently in progress, using the developed sensors and external probes to measure the corrosion rate of the embedded rebar.

## Figures and Tables

**Table 1.** Chemical composition of the carbon steel and of the stainless steel.

**Elements in %**	**C**	**Si**	**Mn**	**P**	**S**	**Cr**	**Mo**	**Ni**	**Cu**	**V**	**W**	**N**	**Fe**
**Stainless steel**	0.03	0.4	2	0.03	0.03	17	2	11	0.5	0.06	0.03	0.05	<68
**Carbon steel**	**0.1**	**0.2**	**0.6**	**0.02**	**0.03**	**0.1**	**0.02**	**0.2**	**0.5**	**0.002**	**0.02**	**0.02**	**98**

**Table 2.** Values of *I*_gal_ corresponding to the standard values of *I*_corr_, indicative of the steel corrosion condition.

**Steel condition**	***I*_corr_** **(μA cm^−2^)**	***I*_gal_** **(nA cm^−2^)**
passive state	<0.1	<0.14
high active corrosion	>1	>1
